# PD-1^+^ T lymphocyte proportions and hospitalized exacerbation of COPD: a prospective cohort study

**DOI:** 10.1186/s12931-024-02847-6

**Published:** 2024-05-24

**Authors:** Hong Xue, Xiuyan Lan, Ting Xue, Xuwei Tang, Haitao Yang, Zhijian Hu, Nengluan Xu, Baosong Xie

**Affiliations:** 1 Department of Respiratory and Critical Care Medicine, Provincial School of Clinical Medicine, Fujian Provincial Hospital, Fujian Medical University, No.134 East Street, Fuzhou, Fujian 350001 China; 2 Center of Health Management, Fujian Provincial Hospital, Shengli Clinical Medical College of Fujian Medical University, Fuzhou, Fujian 350001 China; 3 Department of Epidemiology and Health Statistics, Fujian Provincial Key Laboratory of Environmental Factors and Cancer, School of Public Health, Fujian Medical University, 1 Xuefu north Road, Fuzhou, 350122 China; 4 Department of Infectious Diseases, Provincial School of Clinical Medicine, Fujian Provincial Hospital, Shengli Clinical Medical College of Fujian Medical University, No.516 Jinrong South Street, Fuzhou, Fujian 350001 China

**Keywords:** Chronic obstructive pulmonary disease (COPD), Acute exacerbation, Cell death protein 1(PD-1), T lymphocytes, Flow cytometry analysis, Prognosis predict

## Abstract

**Objective:**

To evaluate the predictive value of PD-1 expression in T lymphocytes for rehospitalization due to acute exacerbations of COPD (AECOPD) in discharged patients.

**Methods:**

115 participants hospitalized with COPD (average age 71.8 ± 6.0 years) were recruited at Fujian Provincial Hospital. PD1^+^T lymphocytes proportions (PD1^+^T%), baseline demographics and clinical data were recorded at hospital discharge. AECOPD re-admission were collected at 1-year follow-up. Kaplan-Meier analysis compared the time to AECOPD readmissions among groups stratified by PD1^+^T%. Multivariable Cox proportional hazards regression and stratified analysis determined the correlation between PD1^+^T%, potential confounders, and AECOPD re-admission. ROC and DCA evaluated PD1^+^T% in enhancing the clinical predictive values of Cox models, BODE and CODEX.

**Results:**

68 participants (59.1%) were AECOPD readmitted, those with AECOPD readmission exhibited significantly elevated baseline PD-1^+^CD4^+^T/CD4^+^T% and PD-1^+^CD8 + T/CD8 + T% compared to non-readmitted counterparts. PD1^+^ T lymphocyte levels statistically correlated with BODE and CODEX indices. Kaplan-Meier analysis demonstrated that those in Higher PD1^+^ T lymphocyte proportions had reduced time to AECOPD readmission (logRank *p* < 0.05). Cox analysis identified high PD1^+^CD4^+^T and PD1^+^CD8^+^T ratios as risk factors of AECOPD readmission, with hazard ratios of 1.384(95%CI [1.043–1.725]) and 1.401(95%CI [1.013–1.789]), respectively. Notably, in patients aged < 70 years and with fewer than twice AECOPD episodes in the previous year, high PD1^+^T lymphocyte counts significantly increased risk for AECOPD readmission(*p* < 0.05). The AECOPD readmission predictive model, incorporating PD1^+^T% exhibited superior discrimination to the Cox model, BODE index and CODEX index, AUC of ROC were 0.763(95%CI [0.633–0.893]) and 0.734(95%CI [0.570–0.899]) (DeLong’s test *p* < 0.05).The DCA illustrates that integrating PD1^+^T% into models significantly enhances the utility in aiding clinical decision-making.

**Conclusion:**

Evaluation of PD1^+^ lymphocyte proportions offer a novel perspective for identifying high-risk COPD patients, potentially providing insights for COPD management.

**Trial registration:**

Chinese Clinical Trial Registry (ChiCTR, URL: www.chictr.org.cn/), Registration number: ChiCTR2200055611 Date of Registration: 2022-01-14.

**Supplementary Information:**

The online version contains supplementary material available at 10.1186/s12931-024-02847-6.

## Introduction

Chronic obstructive pulmonary disease (COPD) was a preventable and manageable respiratory condition characterized by persistent airflow limitation, impacting around 9–10% of elderly individuals [1–3]. Acute exacerbation of COPD (AECOPD) could occur during the natural course of COPD and is related to disease progression [4]. For affected individuals, hospitalization due to AECOPD significantly influenced physical functioning and health-related quality of life.Moreover, it escalated the likelihood of hospital readmission and mortality [3, 4].

Given the substantial population afflicted by this condition, prognostic evaluation in AECOPD is pivotal for devising effective therapeutic strategies [5]. Many models comprised retrospective analysis of routinely collected data such as baseline demographics, baseline severity of disease or indices of hospital admission severity and therefore do not inform clinicians on how prognostic risk might be influenced [6, 7]. Some widely used indices including BODE and its modifications, ADO, DOSE, CODEX, COTE, still had limited accuracy in predicting AECOPD [7].

Due to respiratory infections and environmental exposures potentially triggering AECOPD [8], Cho et al. highlighted immunosenescence as a critical mechanism in COPD development [9]. The immunosenescence lead T cells to dysfunction with metabolic and epigenetic changes, finally leading them to senescence, or even apoptosis [10]. Programmed cell death protein 1 (PD-1) expressed on T cells surface, indicating their functional status [11]. Elevated PD-1 + T lymphocytes in COPD patients [12] promoted interest in their potential involvement in COPD development and progression. Previous research had reported increased PD-1 expression in lung cancer tissues of individuals with concomitant COPD, influencing the effectiveness of PD-1 inhibitors [13, 14]. The severity of COPD correlates positively with CD8^+^ T cells expressing PD-1/TIM-3 (T cell immunoglobulin and mucin-domain-containing molecule-3) [14]. Blocking PD-1 reduced lung damage and neutrophilic inflammation in experimental COPD [15]. Building on these, we regard PD-1 as a potential biomarker for predicting AECOPD readmission, offering a novel perspective.

Our study’s aim was to assess the prognostic significance of PD-1^+^ T lymphocytes in COPD patients. Given PD-1’s role as an immunosenescence marker, we hypothesized that high proportions of PD-1^+^ T lymphocytes would be associated with an elevated AECOPD readmission risk.

## Methods

### Participants

Participants aged 45 to 85, were recruited at discharge following hospitalization for COPD diagnosis given by clinical physicians from the Department of Respiration and Critical Care Medicine at Fujian Provincial Hospital, from 2022 January to August. According to the “Guidelines for the Diagnosis and Treatment of Chronic Obstructive Pulmonary Disease (2021 Revision) [16], the criteria for discharge are as follows: (1) Clinical symptoms and ABG (Arterial Blood Gases) are stable for 24 to 48 h or more, and the primary physician deems the patient suitable for home care, with the degree of dyspnea not affecting daily activities; (2) The patient fully understands and is able to correctly use inhaled medications; (3) Must be able to walk indoors; (4) Serological tests for infection-related indicators return to normal (Procalcitonin < 0.25ng/ml or last C-reactive protein < 5 mg/ml); (6) Follow-up and home care plans are properly arranged. Only those who meet the above conditions and provide informed consent will be screened and included in the cohort study. This study adhered to the principles of Helsinki Declaration. All participants were derived from a cohort study registered on Chinese Clinical Trial Registry(https://www.chictr.org.cn/) (ID: ChiCTR2200055611) and signed informed consent forms. Ethical approval was obtained from the Ethics Committee of Fujian Provincial Hospital (Approval No: K2019-01-003, K2021-09-033).

**Fig. 1:**
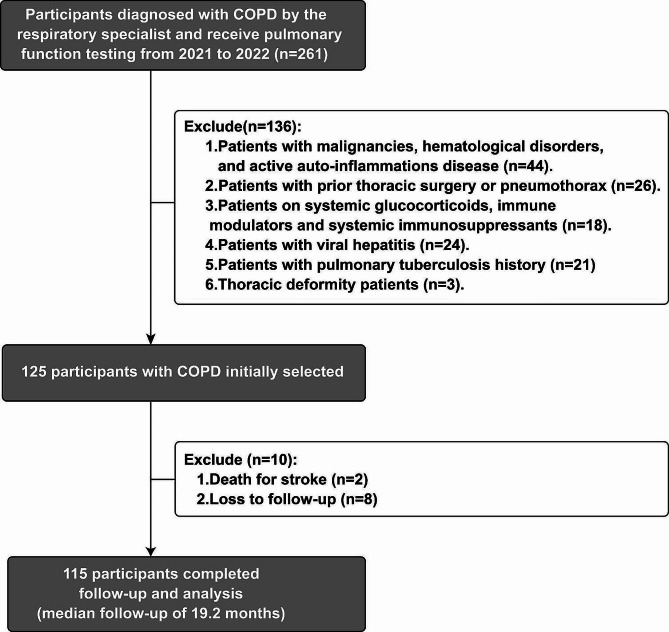
Research Flowchart

The study flow chart is shown in Fig. 1. Inclusion criteria required participants to provide complete blood samples and clinical data, and to consent to follow-up. Exclusion criteria covered malignancies, pregnant or lactating women, active inflammations, diseases necessitating oral corticosteroids/immunosuppressive drugs for over 2 weeks, prior lung surgeries/transplants, blood disorders, and dialysis. All participants received inhaled pharmacological treatment at study commencement, adhering to Chinese guidelines for the diagnosis and management of COPD (version 2021) [16] and the discretion of physicians.

### Measurement

All the measurements were collected within 24 h before hospital discharge, including demographic variables, Body Mass Index (BMI), self-reported AECOPD hospital admissions in previous year, respiratory disability (Medical Research Council (MRC) dyspnea score, Charlson co-morbidity index [17], self-reported domestic oxygen therapy status and medication profiles. The 6-minute walk test(6MWT) were measured. The BODE index for each patient was calculated using the methodology proposed by Celli [18] et al., while the CODEX index was calculated following the Working Group on COPD of the Spanish Society of Internal Medicine guidelines [19].

According to the American Thoracic Society/European Respiratory Society guidelines [20], the lung function of participants were evaluated by MasterScreen lung function (Jaeger, Germany). Evaluated parameters included: (1) Forced Vital Capacity (FVC); (2) Forced expiratory volume in 1 s (FEV1); (3) FEV1/FVC ratio; (4) FEV1 as a percentage of predicted value (FEV1%pred).

The flow cytometry detection protocol for this study was established based on previous research [21, 22].Peripheral blood mononuclear cells (PBMCs) were obtained from bloodstream by Ficoll-Hypaque density gradient centrifugation and cryopreserved in liquid nitrogen. Following thawing, cells were cultured overnight in RPMI 1640 medium with 10% FBS, 2 mM glutamine, and 50 mg/mL gentamycin. Viability, determined by Trypan blue staining, ensured over 90% viable suspensions (see in supplement data). Subjects’ 100 µl PBMCs in PBS underwent CD3-APC, CD4-V450, CD8-FITC and PD-1-PE-Cy7 antibody (BD Biosciences) treatment as instructions. PD-1 antibody was used to detect PD-1 expression on the surface of PBMCs, while CD16 and CD19 were used to exclude interference from PD-1 on the surface of NK cells and B cells. Resuspended cells were analyzed via a Cytomics FC500 flow cytometer. FlowJo version 10 (FlowJo LLC, Ashland, OR USA) conducted data analysis.

### Follow-up protocol

After enrollment, patients were surveyed via telephone or questionnaire. Data included demographics, diagnosis, treatments, AECOPD hospitalizations, timelines, and survival status, and were systematically organized and recorded. The primary outcome was AECOPD hospitalizations during the 1-year follow-up. Hospital readmissions were identified through patient recall and further confirmed by cross-referencing with hospital databases and records. Patients with AECOPD re-admission underwent a cardiac ultrasound during hospitalizations to ascertain pulmonary hypertension (PH). Figure 1 showed the study flow chart.

### Statistical analysis

Continuous variables were expressed as the mean ± standard deviation(SD) or median, while categorical variables as counts and percentages. Normality was assessed with the Shapiro-Wilk test. For normal data, Brown-Forsythe and Welch’s ANOVA and Dunnett’s test were used; non-normal data underwent Kruskal-Wallis ANOVA and Dunn’s tests. Fisher’s test compared categorical variables, setting significance at *P* < 0.05. Coefficient of correlation was calculated using Pearson and Spearman coefficient for normal and non-normal data respectively. The Kaplan-Meier method was employed for univariate analysis of AECOPD re-admission time, with Log-rank test significance level set at *p* < 0.05. Collinearity was judged via variance inflation factor (VIF), VIF > 3 led to exclusion for multivariable analysis. Multivariable Cox proportional hazard regression model (Cox model) and stratified analysis was conducted for risks factors AECOPD re-admission in 1-year.

The area under curves (AUC) of Receiver operating characteristic were generated to assess the predictive values of models with or without PD-1 T cells ratios. The comparison was processed with DeLong’ s test. Decision curve analysis (DCA) was utilized to assess the potential clinical significance of adding the PD1 + T lymphocytes proportions to Cox model and traditional prediction models such as BODE and CODEX. All data were visual analyzed by Origin 2022 and R v4.2.3.

## Results

### Baseline characteristics

Baseline data from 125 patients were collected. Eight lost to follow-up, and two died (stroke or COVID-19). Ultimately, 115 patients were included, with a median follow-up of 19.2 (IQR 13.3–27.2) months (Fig. 1). At the follow-up endpoint, peripheral blood samples were re-collected, subject to patient consent, using established protocols. Finally, 30 patients (15 with AECOPD re-admissions and 15 without (stable COPD)) were selected based on age and gender parity, and their blood samples were included in the subsequent analysis phase. Table 1 and Supplemental Table S1 summarizes baseline demographics and clinical characteristics of the cohort. Recruitment ensured an equal distribution in age, smoking status, lung function, and inhaled medication usage. Out of these, 61 received long-term home oxygen therapy (LTDOT), while 54 did not. Notably, 49 cases (45.8%) experienced AECOPD readmission within 1 year.

**Table 1:** Characteristics of participants at baseline

		Total	PD1^+^ CD4^+^ T level			PD1^+^ CD8^+^ T level		
			High	Low	*p*	High	Low	*p*
		(*n* = 115)	(*n* = 48)	(*n* = 67)		(*n* = 52)	(*n* = 63)	
Age(years)		71.83 ± 6.0	72.95 ± 5.6	71.03 ± 5.8	0.39	72.74 ± 6.3	71.08 ± 5.7	0.39
Males (n (%))		95(82.6)	40(83.3)	55(82.1)	0.50	43(82.7)	52(82.5)	0.49
BMI(Kg/m2)		24.5 ± 4.71	24.4 ± 4.6	24.6 ± 4.9	0.28	24.1 ± 4.8	24.8 ± 4.7	0.28
Smoke History(pack-year)		63.9 ± 32.8	65.9 ± 34.1	62.48 ± 30.6	0.56	65.9 ± 31.1	62.5 ± 34.8	0.56
FEV1/FVC(%)		50.08 ± 8.7	48.75 ± 9.3	51.05 ± 8.3	0.42	49.20 ± 9.3	51.44 ± 8.6	0.42
GOLD grade	1∽2	49(42.6)	19(39.6)	30(44.8)	0.36	21(40.4)	28(44.5)	0.40
	3∽4	66(57.4)	29(60.4)	37(55.2)		31(59.6)	35(47.6)	
mMRC scores	0–1	52(45.2)	21(43.7)	31(46.3)	0.53	22(42.3)	30(47.6)	0.35
	≥ 2	63(54.8)	27(56.3)	36(53.7)		30(57.7)	33(52.3)	
CAT scores	≤ 10	19(16.5)	8(16.7)	11(16.4)	0.43	8(15.4)	11(17.5)	0.43
	11∽20	43(37.4)	18(37.5)	25(37.3)		19(36.5)	24(38.1)	
	21∽30	38(33.0)	16(33.3)	22(32.8)		18(34.6)	20(31.7)	
	≥ 31	15(13.0)	6(12.5)	9(13.4)		7(13.5)	8(12.7)	
PaO2/FiO2		295.7 ± 30.9	292.1 ± 35.7	298.3 ± 30.1	0.38	291.8 ± 29.7	298.9 ± 32.2	0.30
Inhale drugs	Bronchodilators, n(%)	51(44.3)	23(47.9)	28(59.6)	0.32	22(42.3)	29(46.1)	0.42
	ICS + Bronchodilators, n(%)	64(55.7)	25(52.1)	39(58.2)		30(57.7)	34(53.9)	
Number of AECOPD in previous year		2(1,3)	2.5(1,3)	2(1,3)	0.20	2.5(1,3)	2(1,3)	0.20
LTDOT, Yes(n)		61(53.0)	29(60.4)	32(47.8)	0.18	28(53.8)	33(52.3)	0.47
6MWT(m)		331.3 ± 33.2	323.0 ± 32.5	337.2 ± 34.0	0.13	324.2 ± 31.9	337.1 ± 34.4	0.11
age-adjusted Charlson Comorbidity Index, aCCI		4(2,5)	3.5(2,5)	4(2,5)	0.16	4(2,5)	4(2,5)	0.16
AECOPD hospital readmission		68(59.1)	30(62.5)	38(56.7)	0.33	33(63.4)	35(55.6)	0.39

### Lymphocyte subpopulation alterations in COPD patients

**Fig. 2:**
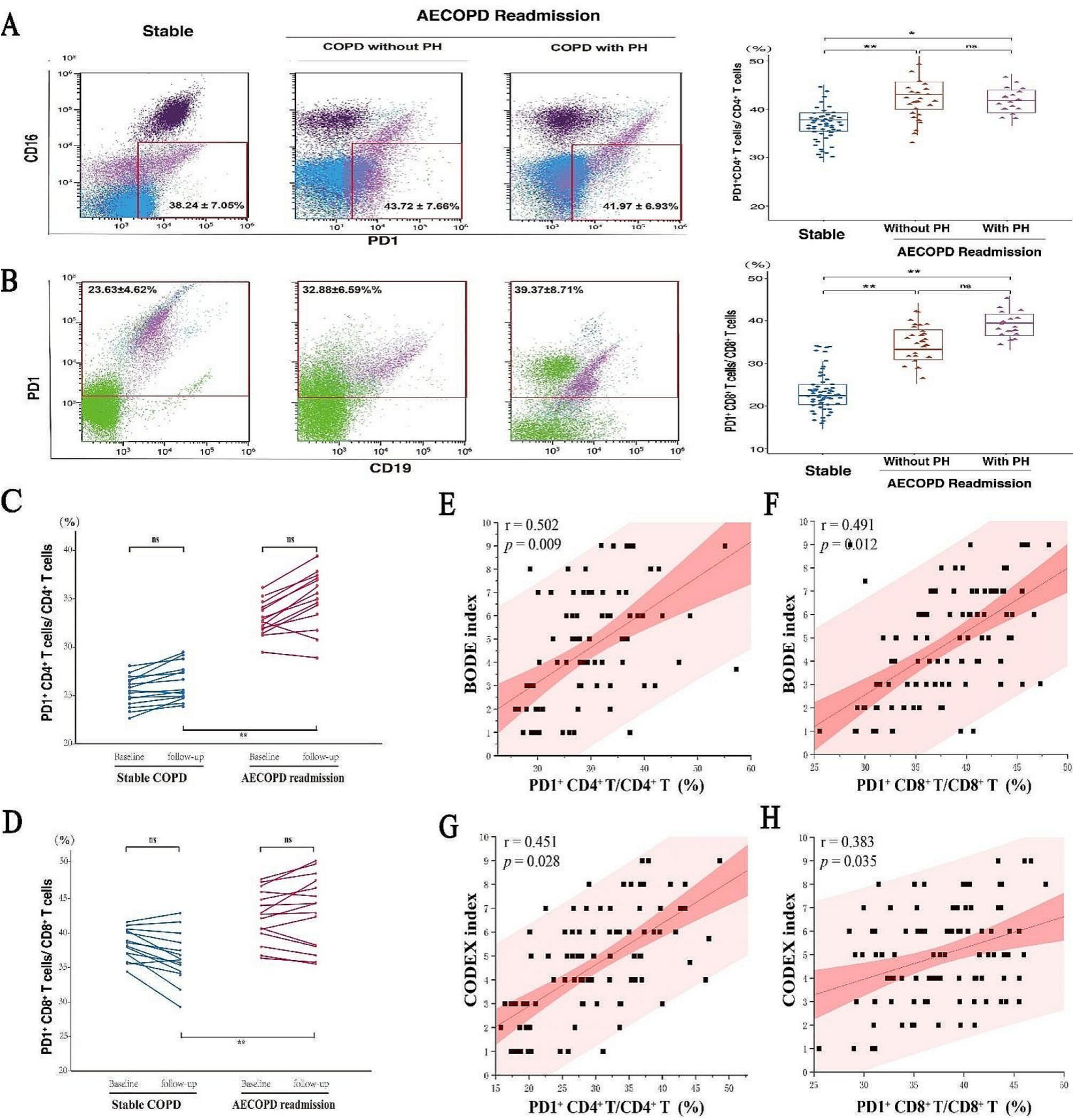
Analysis of changes and correlations in PD1 + T lymphocytes among COPD participants with different prognosis. **A&B.** Baseline differences in PD-1^+^CD4^+^T and PD-1^+^CD8^+^T lymphocyte subgroups between COPD participants with and without AECOPD re-admissions (*: *p* < 0.05, **: *p* < 0.01). **C&D.** Baseline and follow-up expression profiles of PD-1^+^CD4^+^T and PD-1^+^CD8^+^T lymphocyte subgroups in COPD participants (*: *p* < 0.05, **: *p* < 0.01). **E&F** Correlation analysis of PD-1^+^CD4^+^T and PD-1^+^CD8^+^T lymphocyte subgroups with the BODE index in COPD participants. **G&H.** Correlation analysis of PD-1^+^CD4^+^T and PD-1^+^CD8^+^T lymphocyte subgroups with the CODEX index in COPD participants

In our cohort, significant disparities of PD1 + T cellular phenotypes were observed in baseline. Specifically, participants experiencing AECOPD re-admission, regardless of PH coexistence, showed a significant increase in the proportion of PD-1^+^CD4^+^T lymphocytes among CD4^+^T lymphocytes (PD-1^+^CD4^+^T/ CD4^+^T%) and PD-1^+^CD8^+^T lymphocytes among CD8^+^T lymphocytes (PD-1^+^CD8^+^T/ CD8^+^T%) at baseline, compared to those without AECOPD re-admissions. Our findings revealed no significant differences in PD-1^+^CD4^+^T/ CD4^+^T% and PD-1^+^CD8^+^T/ CD8^+^T% among individuals experiencing AECOPD re-admission, regardless of the presence of concurrent pulmonary hypertension (PH)(Figure 2A, B).

Post-one-year follow-up assays revealed that AECOPD re-admissions participants exhibited higher proportions of PD-1 + CD4 + T/CD4 + T% and PD-1 + CD8 + T/CD8 + T% compared to those without AECOPD re-admission, consistent with baseline data. These sustained elevations, coupled with significant group differences, *underscored* the stability of PD1 + T lymphocytes as a distinguishing biomarker(Figure 2C, D).

.

### Relationship between PD1 T lymphocytes proportions and COPD prognostic indices

A statistically significant moderate positive correlation was observed between the baseline PD-1 + CD4 + T/CD4 + T% and the BODE index (*r* = 0.502, *p* = 0.009). Similarly, a statistically significant moderate positive correlation was observed between baseline PD-1 + CD8 + T/CD8 + T% and the BODE index (*r* = 0.491, *p* = 0.012). (shown in Fig. 2E, F). As the inclusion of comorbidity assessment in the CODEX index was also a common prognostic indicator for COPD, the PD-1^+^CD4^+^T/ CD4^+^T% (*r* = 0.451) and PD-1^+^CD8^+^T/ CD8^+^T% (*r* = 0.383) at baseline were demonstrated the moderate positive correlations with patients’ CODEX indices, with statistically significant(*p* < 0.05). (Fig. 2G, H).

### Kaplan-Meier analysis for COPD patient survival

**Fig. 3:**
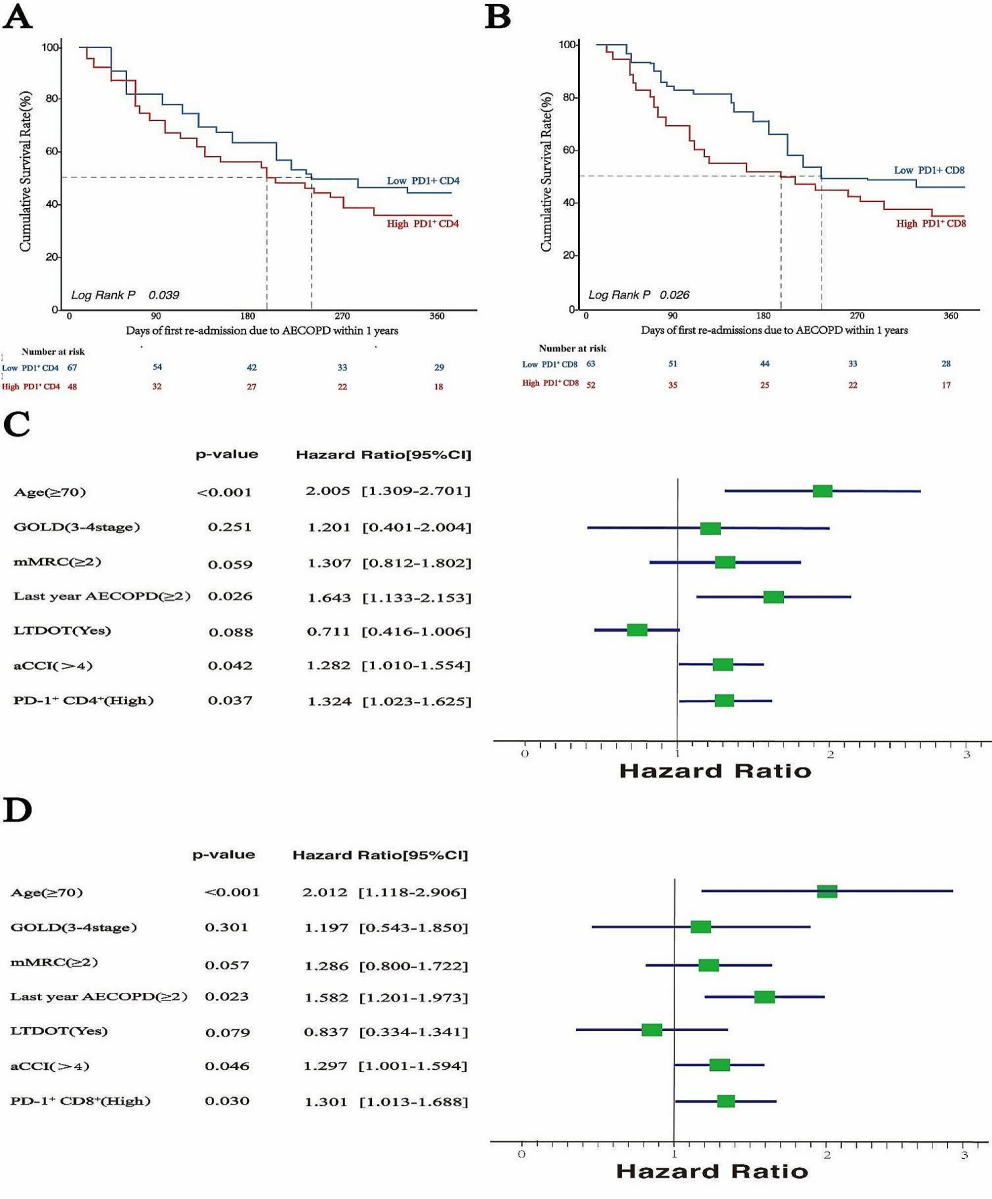
Impact of PD1 + T lymphocyte proportions on AECOPD re-admission **A&B** Kaplan-Meier curves depicting time to AECOPD re-admissions across various PD1 + CD4 T and PD1 + CD8 T lymphocyte proportion groups. **C&D** Multivariable Cox proportional hazard regression analysis identifying risk factors for AECOPD re-admission

Univariate Kaplan-Meier analysis notably identified that subjects aged over 70, with GOLD grades 3–4, mMRC scores ≥ 2, no LTDOT, and high PD1 + T lymphocyte proportions, had statistically significantly shorter times to first AECOPD hospitalization post-enrollment. Gender, aCCI, and 6MWT showed no significant impact. Comprehensive results were outlined in Table 2. Participants were divided into two groups based on median PD-1^+^CD8^+^T/CD8^+^T% and PD-1^+^CD4^+^T/CD4^+^T%: High and Low PD1^+^CD8^+^T, and High and Low PD1^+^CD4^+^T, respectively. In the Kaplan-Meier survival curves (Fig. 3A, B), the unadjusted data demonstrated the differences in PD1 + T lymphocyte proportions and AECOPD rehospitalization.

**Table 2:** Kaplan-meier survival analysis results for participants

Variable		n	Median AECOPD re-admission times in 1 year(day)	Log-Rank	
				x^2^	p
Age	≥ 70years	55	191.8	21.4	< 0.001
	<70years	60	244.7		
Sexual	Male	95	218.6	0.98	0.07
	Female	20	238.4		
GOLD grade	1–2	49	234.7	3.11	0.05
	3–4	66	212.2		
mMRC scores	0–1	52	240.4	4.99	0.03
	≥ 2	63	206.3		
Inhale drugs	Bronchodilators	51	232.4	0.83	0.24
	ICS + Bronchodilators	64	213.2		
Number of AECOPD in previous year	0–1	73	234.9	6.61	0.02
	≥ 2	42	198.8		
LTDOT	Yes	61	230.0	1.08	0.21
	No	54	210.5		
PD1 + CD4 T level	Low	67	235.6	4.08	0.03
	High	48	202.3		
PD1 + CD8 T level	Low	63	233.9	3.59	0.04
	High	52	207.0		
aCCI	0–3	66	232.1	0.77	0.29
	≥ 4	47	216.6		
6MWT	< 350 m	47	210.0	0.61	0.26
	≥ 350 m	68	229.8		

### Cox regression analysis indicate PD1 + T lymphocyte ratio as the risk factors for AECOPD readmission

Owing to collinearity (VIF > 3), 6WMT was omitted from the Cox regression analysis. Considering the interdependence between PD-1^+^CD4^+^T/CD4^+^T% and PD-1^+^CD8^+^T/CD8^+^T% ratios, each was separately integrated into the Multivariable Cox proportional hazard regression analysis. The analysis identified multiple independent risk factors for AECOPD re-admission within one year, such as age over 70, more than two AECOPD episodes in the previous year, a high aCCI index, and elevated levels of PD1 + CD4 + T and PD1 + CD8 + T. LTDOT was identified as a protective factor. Participants with high PD1 + CD4 + T levels showed a 38.4% increased risk of AECOPD re-admission (Hazard Ratio [HR] 1.324, 95%CI [1.023–1.625]). Similarly, High PD1 + CD8 + T participants encountered a 40.1% higher risk of AECOPD re-admission (HR 1.301, 95% CI [1.013–1.688]). (Fig. 3C, D)

### Interactive stratified analysis of PD1 + T lymphocytes with other risk factors in cox analysis

**Fig. 4:**
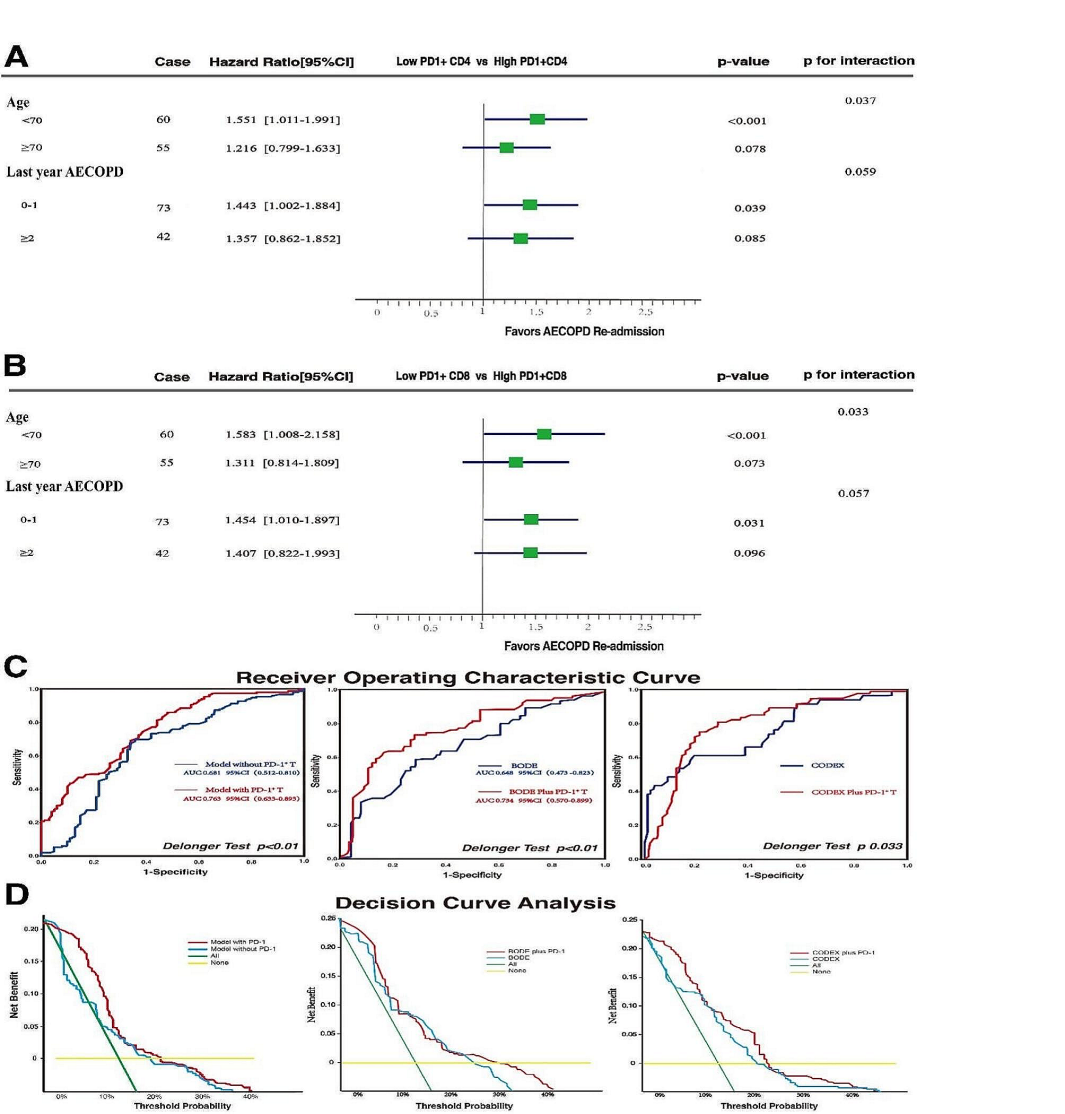
Impact of PD1 + T lymphocyte proportions on AECOPD re-admission predictive model. **A&B** Hazard Ratio stratified by age and last year AECOPD (Cox proportional hazards regression). **C** ROC curve comparison Predictive Performance of AECOPD Readmissiont in Models with and without of PD-1 T Lymphocyte Proportions **D** DCA curve illustrates the utility in aiding clinical decision-making of models predicting AECOPD readmission with and without PD-1 + T lymphocyte proportions

The results in Fig. 4A and B indicated an interaction between the proportions of PD1 + CD4 + T cells, PD1 + CD8 + T cells, and age on the recurrence of AECOPD (interaction *p* = 0.037, 0.033). Among individuals younger than 70 years old, the increased percentages of PD1^+^CD4^+^ and PD1^+^CD8^+^ cells at discharge in COPD patients were significantly associated with a higher risk of recurrent AECOPD within 1 year of rehospitalization, with risks elevated by 55.1% and 58.3%, respectively (*P* < 0.001). However, in the age > 70 group, participants with high PD1^+^CD4^+^ T or PD1^+^CD4^+^ T didn’t exhibit significantly increased risk of AECOPD readmission (HR 1.216, 95% CI [0.799–1.633]).

Figure 4A and B demonstrated that among patients with fewer than two episodes of AECOPD in the previous year, those discharged with increased percentages of PD1^+^CD4^+^ or PD1^+^CD8^+^ cells faced a significantly higher risk of readmission due to AECOPD within one year, with risk increases of 44.3% and 45.4%, respectively (*P* < 0.05). Additionally, no statistical significance was observed in the subgroup experiencing at least two AECOPD incidents in the previous year. On the other hand, the proportions of PD1 + CD4 + T cells and PD1 + CD8 + T cells did not show a significant interaction effect on the recurrence of AECOPD based on previous AECOPD episodes (interaction *p* = 0.059, 0.057).

### Incorporating the PD1 + T lymphocyte ratios enhanced the predictive values of the models

Inclusion of PD1 + T lymphocyte ratios in the models notably enhanced predictive performance, as evidenced by ROC analysis. The Cox model with these ratios achieved an AUC of 0.763 (95% CI: [0.633–0.899]), superior to the 0.681 AUC (95%CI: [0.512–0.810]) of the model excluding them. Incorporating these ratios into BODE and CODEX indices also improved AUC, with Delong’s Test confirming these enhancements (Fig. 4C). Clinical Decision Curves for both models *demonstrated* that, across most thresholds, the model incorporating the PD1^+^ T cell ratios *offered* superior clinical decision-making utility compared to the model without these ratios (Fig. 4D).

## Discussion

Our prospective study suggested that the elevated proportion of PD1^+^ T lymphocytes in COPD patients at hospital discharge may independently predict an increased risk of AECOPD readmission in the next year and may have value in the risk stratification of patients surviving a hospitalization for AECOPD.

COPD is a highly heterogeneous condition, making the assessment of its prognosis a pivotal concern in clinical practice. Influenced by respiratory pathogen infections and exposure to harmful airborne particles, alteration in T lymphocyte function played a crucial role in the changing immune landscape of COPD patients [8]. . Variations in the expression of the PD-1 surface antigen were often associated with functional changes in T lymphocytes. It was observed that in lung cancer patients with concomitant COPD, the co-expression of PD-1/TIM-3 on CD8^+^T lymphocytes positively correlated with lung function decline [13]. Moreover, the favorable clinical outcomes usually associated with T lymphocytes in COPD patients were compromised [23]. These findings suggested a potential correlation between increased T lymphocytes PD-1 expression and COPD. A study revealed higher proportions of PD-1^+^CD4^+^ and PD-1^+^CD8^+^ T lymphocytes in the peripheral blood of COPD patients compared to non-COPD individuals after in vitro stimulation [24]. Researchers noted that, before stimulation, the proportions of PD-1^+^CD4^+^ and PD-1^+^CD8^+^ T lymphocytes in total lymphocytes differed between COPD and non-COPD patients, but these differences were not statistically significant [24]. Inconsistently with this report, our focus was on the proportion of PD-1 + T lymphocytes within CD4^+^ and CD8^+^ lymphocytes, this discrepancy might be attributed to potential alterations in the overall level of T lymphocytes in COPD patients compared to healthy individuals.

Our study employed methodologies in line with prior clinical investigations [25–27]. Furthermore, assessments conducted one-year post-follow-up among patients indicated that PD1^+^ T cell levels were still elevated in individuals with frequent AECOPD episodes, compared to those without AECOPD readmissions, consistent with baseline data. The sustained elevations, along with notable group differences, highlight PD1^+^ T lymphocytes as a discriminative biomarker. They exhibited consistent expression patterns unaffected by patient condition changes, making them ideal for clinical detection. Additionally, this findings supported our research hypothesis: the immunological changes linked to PD1^+^ T lymphocytes may lead to recurrent AECOPD readmissions in patients, thus not merely reflecting the AECOPD readmission outcome.

COPD was increasingly acknowledged as a condition linked to the aging of the lungs [28]. Pulmonary aging constitutes a multifaceted process related to immunosenescence [29]. Immunosenescence correlated with an increased expression of aging markers in crucial phenotypic transitions within T lymphocytes [29]. Throughout the immunosenescence, T lymphocytes gradually transitioned into a phenotypic of functional dysregulation, undergoing metabolic reprogramming and epigenetic reshaping, consequently leading to senescence, aging, and even apoptosis [10]. The augmented presence of exhausted T cells was frequently regarded as a principal hallmark of immunosenescence [11]. Although PD-1 was recognized as a marker for exhausted T cells in the contexts of tumors and autoimmune diseases [30], its involvement in immune dysfunction associated with COPD had been inadequately investigated [31]. Continuous PD-1 activation limited T cell effector function, notably cytotoxic activity. After being exposed to chronic antigens and PD-1 stimulation, T cells progressively lost effector functions, compromising infection control [32, 33]. The researcher noted a significant interestingly correlation: COPD combined with anti-PD-1 or anti-PD-L1 therapy correlated with prolonged progression-free survival in lung cancer patients [13]. COPD patients often happened chronic infections and microbial imbalances, leading to local lung inflammation and altered cytokines, these factors affected cell-to-cell interactions, potentially upregulating PD-1 expression in activated T cells [34]. Hence, alterations in lymphocyte PD-1 expression might indirectly mirror compromised immunity in COPD. Our monitoring and follow-up observations in COPD patients provided evidence supporting this notion.

The GOLD reported describes that up to 90% of COPD patients had an average pulmonary artery pressure (mPAP) > 20 mmHg [35]. Pulmonary hypertension(PH) was also frequently a comorbidity in severe COPD [36], recognized as a clinical prognostic factor indicating not only impaired lung function but also affecting right heart function [37]. Immune dysregulation was a common feature of PH [38, 39], and previous research had confirmed the overexpression of PD-1 on peripheral blood lymphocytes in patients with idiopathic pulmonary arterial hypertension and its association with poor clinical parameters [40]. Given the crucial clinical significance of pulmonary hypertension in COPD management, we conducted a subgroup analysis in our study focusing on COPD patients with concomitant PH. Our data indicated that COPD participants with concomitant PH do not exhibit differences compared to those without PH. This complexity might be attributed to the intricate pathogenic mechanisms underlying PH in COPD patients, where PD1^+^ T cells might not be the sole cells influencing vascular function. It might be also possible that the sample size reduction due to stratification based on PH status might have precluded the demonstration of statistical differences at the current stage.

Our study identified age, prior AECOPD occurrences, and PD-1^+^ T cell proportion as high-risk factors for AECOPD readmission within one year in a multifactorial COX model.In the revised manuscript, we conducted an interaction analysis between PD1^+^ T cell proportion and age, delineating the interaction effects on P-values in Fig. 4A and B.The results indicated an interaction between PD-1^+^ T cells and age concerning AECOPD recurrence (*p* = 0.037). In individuals under 70 years old, an increased percentage of PD-1^+^CD4^+^ or PD-1^+^CD8^+^ cells in discharged COPD patients was significantly associated with AECOPD recurrence within one year after discharge, with risks increasing by 55.1% and 58.3% respectively (*p* < 0.001). Age had correlated with response to anti-PD1 drug, reflecting age-related differences in regulatory T-Cell populations [41]. Single-cell RNA sequencing in both young and elderly animals has shown that aging lead to significant remodeling and phenotypic reprogramming of T-cell landscapes [42]. The COVID-19 pandemic had underscored that the accelerated immune ageing was associated with respiratory disease progression [43]. PD-1 had been recognized as a marker of immune cell aging, which drived systemic aging [44, 45]. Interventions targeting immune aging to enhance vigorous immune cells could mitigate the aging process [44]. Therefore, our study suggests that the increase in PD-1-related T cell proportions in COPD patients under 70 years old might indicated an accelerated immune aging relative to physiological aging in this population, rendering them more susceptible to AECOPD exacerbations and increased hospitalization risk. PD1 might serve as a marker of immune aging in such patients, holding potential value for clinical monitoring. Evaluating COPD prognosis was intricate, with AECOPD as a key clinical indicator. Our study revealed shorter AECOPD onset times might related with elevated PD-1 T^+^ cells ratio. McKendry’s in vitro study on influenza virus infection of excised lung tissue demonstrated that impaired antiviral function in CD8^+^T cells of COPD patients compared to those without COPD, which was attributed to the upregulation of PD-1 expression [46]. Based on these foundational studies and current data, we hypothesized that upregulation of PD-1 in COPD assciated to phenotypic changes in T lymphocytes, diminishing their reactivity to novel antigens and increasing the risk of infection-triggered AECOPD. However, further clinical experiments and in-depth research are required to elucidate the role of PD-1^+^ lymphocytes in COPD patients, representing a highly valuable research direction.

### Strengths and limitations

A key strength of our study is its status as one of the few prospective studies examining the association between objectively measured immune functional status at hospital discharge and patient outcomes. We conscientiously conducted a comprehensive assessment at a single time-point (hospital discharge) to evaluate potential confounding factors’ significance and collected reliable follow-up data on hospital admissions confirmed by medical records. Our analysis *proposed* PD-1^+^T lymphocyte proportions as a novel COPD biomarker for COPD prognosis. The integration of PD-1^+^T lymphocyte proportions into predictive models enhanced clinical decision-making. Monitoring PD-1 levels in T lymphocytes offers promising clinical insights.

Due to the limitations of a single-center study, it is essential to validate these results in independent cohorts from diverse healthcare systems with different models of inpatient care and post-discharge follow-up. Another research area worth exploring is whether routine monitoring of PD1 + T lymphocytes during COPD management could impact decision-making regarding discharge timing.

## Conclusion

Elevated PD1 + T lymphocyte ratios may serve as an independent risk factor for 1-year AECOPD readmission. As a non-invasive measure, we suggest that routine measurement at hospital discharge could offer clinicians valuable information for planning post-discharge care and support.

### Electronic supplementary material

Below is the link to the electronic supplementary material.

Supplementary Material 1

## Data Availability

The datasets used and/or analyzed during this study are available from the corresponding author on reasonable request.

## Abbreviations

COPD: Chronic Obstructive Pulmonary Disease
PD-1: Cell Death Protein-1
AECOPD: Acute Exacerbation of COPD
BMI: body mass index
FEV1: forced expiratory volume in 1 s
MRC: Medical Research Council
CAT: COPD assessment test
LTDOT: long-term home oxygen therapy
6MWT: 6-minute walking test
aCCI: age-adjusted Charson comorbidity index
GOLD: global initiative for chronic obstructive lung disease
PH: pulmonary hypertension
ROC: Receiver operating characteristic
AUC: Area under Curve
BODE: the body-mass, airflow obstruction, dyspnea, and exercise capacity index
CODEX: Comorbidity, Obstruction, Dyspnea, and Previous Severe Exacerbations index
DCA: Decision curve analysis
