# Supplementary material for: PD-1+ T lymphocyte proportions and hospitalized exacerbation of COPD: a prospective cohort study
Source: Respir Res. 2024 May 24;25:218. doi: 10.1186/s12931-024-02847-6 (PMC11127417; doi:10.1186/s12931-024-02847-6)
Supplement: Supplementary file 1 — Supplementary Material 1 [file 12931_2024_2847_MOESM1_ESM.docx]

Supplemental Data

**TABLE S1.**

**Baseline characteristics of whole cohort stratified by Follow-Up Outcome**

**Table 1 Characteristics of participants at baseline**

|  | | **Total** | **Follow-Up Outcome** | | ***p*** |
| --- | --- | --- | --- | --- | --- |
|  |  |  | **Not AECOPD readmitted** | **AECOPD readmitted** |  |
|  |  | **(n=115)** | **(n=47)** | **(n=68)** |  |
| Age(years) | | 71.8±6.0 | 70.9±6.2 | 72.2±5.9 | 0.346 |
| Males, n (%) | | 95(82.6) | 54（81.8） | 41(83.6) | 0.627 |
| BMI(Kg/m2) | | 24.5±4.7 | 24.2±4.7 | 24.9±4.6 | 0.229 |
| Smoke History（pack-year） | | 63.9±32.8 | 64.5±31.1 | 63.5±32.6 | 0.518 |
| FEV1/FVC（%） | | 50.08±8.7 | 51.89±8.9 | 50.09±8.6 | 0.504 |
| GOLD grade | 1-2, n (%) | 49(42.6) | 21(44.7) | 28(41.2) | 0.192 |
|  | 3-4, n (%) | 66(57.4) | 26(55.3) | 40(58.8) |  |
| mMRC scores | 0-1, n (%) | 52(45.2) | 22(46.8) | 30(44.1) | 0.232 |
|  | ≥2, n (%) | 63(54.8) | 25(53.2) | 38(55.9) |  |
| CAT scores | ≤10, n (%) | 19(16.5) | 10(21.3) | 9(13.2) | 0.211 |
|  | 11-20, n (%) | 43(37.4) | 18(38.3) | 24(35.3) |  |
|  | 21-30, n (%) | 38(33.0) | 15(31.9) | 23(33.8) |  |
|  | ≥31, n (%) | 15(13.0) | 5(10.6) | 10(14.7) |  |
| PaO2/FiO2 | | 295.7±30.9 | 299.9±32.8 | 292.8±29.8 | 0.335 |
| Inhale drugs | Bronchodilators, n(%) | 51(44.3) | 20(42.5) | 31(45.6) | 0.263 |
|  | ICS + Bronchodilators, n(%) | 64(55.7) | 29(61.7) | 35(51.5) |  |
| Number of AECOPD in previous year | | 2（1，3） | 1.5(1,3) | 2（1，3） | 0.344 |
| LTDOT, n (%) | | 61(53.0) | 28(59.6) | 33(48.5) | **0.041** |
| 6MWT(m) | | 331.3±33.2 | 339.5±34.3 | 325.6±30.1 | **0.036** |
| Age-adjusted Charlson Comorbidity Index, aCCI | | 4(2,5) | 3.5(2,5) | 4(2,5) | 0.536 |

**Notes:** Data were presented as median (IQR first and third quartiles) or number (percentage)or mean ± SD. P-values were determined by the Mann–Whitney U-test for continuous variables and the chi-square test or Fisher exact test for categorical variables. Values in bold indicate statistical significance at the P < 0.05.

**2.Data of cytometry vitality detection using trypan blue staining before flow cytometry analysis of revived cells**


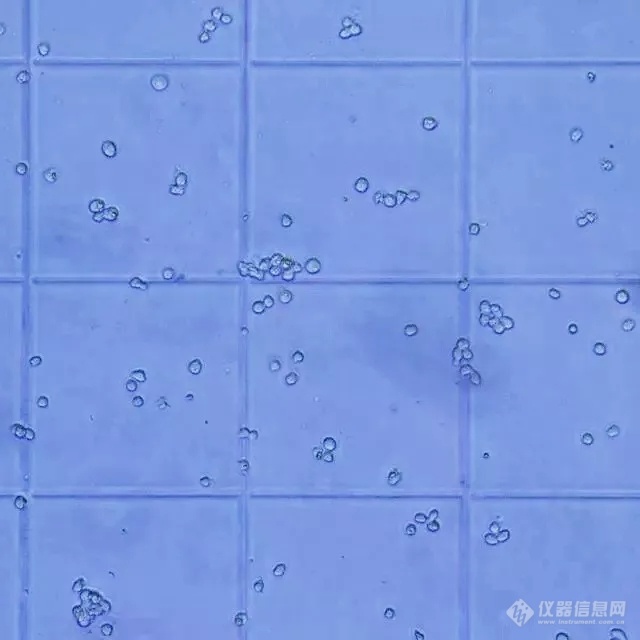


**PBMC Viability: 93.51% (91.43% - 96.75%)**
